# Assessment of Cervical Joint Position Sense and Head Posture in Individuals With Myogenic Temporomandibular Dysfunctions and Identifying Related Factors: A Case‐Control Study

**DOI:** 10.1111/joor.13885

**Published:** 2024-10-20

**Authors:** Ümit Yüzbaşıoğlu, Besime Ahu Kaynak, Serkan Taş

**Affiliations:** ^1^ Department of Therapy and Rehabilitation Toros University, Vocational School of Health Services Mersin Turkey; ^2^ Department of Dental Services Toros University, Vocational School of Health Services Mersin Turkey; ^3^ Department of Physiotherapy and Rehabilitation Toros University, Faculty of Health Sciences Mersin Turkey

**Keywords:** inclinometer, pain, posture, proprioception, ROM, temporomandibular joint disorder

## Abstract

**Background:**

Temporomandibular dysfunctions (TMDs) have the potential to cause changes in cervical muscle strength, muscle endurance and position sense by changing muscle activation patterns, especially as a result of forward head posture. The effects of TMDs on cervical joint position sense (CJPS) and head posture remain controversial.

**Objective:**

The aim of this study was to evaluate the head posture and CJPS of individuals with TMDs and compare them with healthy individuals.

**Methods:**

This research, which was designed as a case‐control study, was concluded with the inclusion of total of 84 participants (42 individuals diagnosed with myogenic TMDs, 42 controls). The assessment of participants included pain severity, neck and jaw functionality and disability, CJPS, head posture and temporomandibular joint (TMJ) range of motion (ROM).

**Results:**

Individuals with TMDs exhibited higher angular deviation in CJPS during flexion and extension (*p* < 0.001). Additionally, individuals with TMDs demonstrated higher TMJ pain, limitation and dysfunction severity, as well as a more limited TMJ ROM (*p* < 0.001). Head posture was similar between groups (*p* > 0.05). There is a significant relationship between VAS‐TMJ with VAS‐cervical, FAI, NDI, JFLS‐8 and TMJ ROM (*p* < 0.05). Moreover, a significant correlation was observed between NDI with FAI and TMJ ROM (*p* < 0.05).

**Conclusion:**

These results indicate that in addition to higher pain severity, disability and lower jaw ROM, CJPS of individuals with TMDs is also negatively affected. Also, parameters related to disability and functionality of cervical and TMJ were significantly correlated. Further studies are needed to determine the factors contributing to these results.

## Graphical abstract

Clinical strategies to improve balance and proprioception may reduce symptoms associated withTMDs and enhance jaw function in patients with TMDs.

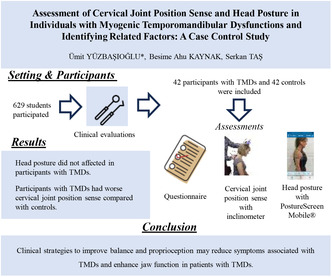

## 1 Introduction

Temporomandibular dysfunctions (TMDs), defines heterogeneous dysfunctions of masticatory muscles and peri‐articular tissues [1]. TMDs has the highest incidence between the ages of 20 and 40 years and affects 15%–20% of adults [2, 3]. TMDs, has a multifactorial aetiology with environmental, social, biological, emotional and cognitive origins [3, 4]. In individuals with TMDs, limitation in temporomandibular joint (TMJ) movements, pain in masseter and temporalis muscles, joint crepitation, headache, neck pain and shoulder pain are among the common symptoms [4].

The stomatognathic system (SS), which is among the cornerstones of postural control, includes TMJ, dental or periodontal tissue and masticatory neuromuscular system [5]. TMJ, forms a functional complex called “cranio‐cervico‐mandibular system” by establishing anatomical, biomechanical physiological and neuronal connections with the cervical region [6]. Changes in the cervical posture have the potential to influence jaw movements and the activation of masticatory muscles, given the muscular and ligamentous connections between the TMJ and the cervical spine [7]. Existing evidence suggests cervical involvement in masticatory muscle pain may arise from two mechanisms: convergence of neck pain inputs on trigeminal motor neurons, leading to increased masticatory muscle activity, and reflexive contraction of masticatory muscles in response to cervical muscle contraction [8]. It is thought that a pathology in any component of the SS can alter the proprioceptive system due to its close functional relationship [6].

It was reported that head–neck posture may affect the development of TMDs and cervical vertebral bone alignment [9]. Olmos et al. [10] concluded that TMDs are associated with forward head posture (FHP). In contrast, Hackney et al. [11], reported that individuals with TMDs have similar degree of FHP compared to healthy controls. Although it is thought that mandibular dysfunction and problems in the neck joint are significantly related, the relationship between head and cervical posture and TMDs is still controversial [9, 10, 11].

Different cervical positions, considered to be one of the components affecting mandibular position and chewing muscle functions [12]. Furthermore, a decrease in strength and endurance of neck muscle was reported in patients with TMDs compared to controls [13, 14]. Electromyography (EMG) studies found that people with myogenic TMDs show higher activity in superficial cervical muscles [15, 16]. It has been reported that FHP inhibits muscle activation patterns by reducing cervical spine endurance, which may affect spinal movement patterns and cause changes in the sense of position [17].

Given the potential effects of alterations on endurance, muscle strength and muscle activation parameters on cervical joint position sense (CJPS) [13, 14, 15, 16] and the fact that relationship between head–neck posture and TMDs is still controversial [9, 10, 11], effects of TMDs on CJPS and head posture remains a mystery. In addition, no study was found in the literature that evaluated CJPS in individuals with TMDs and compared it with healthy controls. The primary aim of this case‐control was to evaluate the head posture and CJPS of individuals with TMDs and compare them with healthy individuals. Secondary aim of the study was to examine the relationship between the deviation in CJPS and changes in head position with the range of motion (ROM) of the TMJ, pain and limitation in jaw functions in individuals with TMDs.

## 2 Material and Methods

### 2.1 Ethics Statement

Ethical approval for this study was obtained by the Toros University Non‐invasive Clinical Research Ethics Committee with decision number (2023/05‐16). Participants gave verbal and written informed consent. Declaration of Helsinki and STROBE statement for case‐control studies were followed by our study [18]. The STROBE checklist is given in Data S1.

### 2.2 Sample Size

Sample size calculation made with G*Power software (Version 3.1.9.7; Heinrich‐Heine‐Universität Düsseldorf, Düsseldorf, Germany) to determine the number of participants to be included in the study. When the mean target angle value in control group was 0.98° with a standard deviation of 1.32°, it was determined that the minimum sample size required to detect the minimum clinically significant difference was 37 individuals for each group with 95% power and 0.05 margin of error [19].

### 2.3 Individuals

A total of 629 students studying at Toros University 45 Evler Campus were administered the Fonseca Anamnestic Index (FAI) between October 2023 and December 2023. Individuals with FAI scores below 15 and above 45 have been invited to participate in the study. The FAI score, reported by Kaynak et al. [20] to have good to excellent test–retest reliability, high internal consistency, and diagnostic accuracy to determine the presence in Turkish populations. FAI score when it is 20 or above indicates the presence of TMDs, while a score of 15 or below indicates the absence of TMDs [20, 21]. Clinical evaluations to determine the presence and type of TMDs were performed by a maxillofacial surgeon with 24 years of professional experience according to the Diagnostic Criteria for TMDs (DC/TMD). After clinical evaluations, individuals with only myogenic TMDs were included. Individuals between the ages of 18 and 45 years who volunteered to participate in the study and who did not have any communication difficulties were included in the study. The study excluded individuals with generalised joint damage affecting the head, neck and shoulder regions, individuals with a history of major trauma, fracture or surgery related to these body regions or undergoing radiotherapy, individuals with a neck disability index score of 25 or higher indicating severe and total disability, individuals diagnosed with cervical disc herniation, radiculopathy or myelopathy, individuals with a history of congenital disease and pregnant individuals. In addition to these criteria, individuals with any neurologic, systemic and cardiopulmonary conditions were also excluded. The study group consisted of individuals who were diagnosed with myogenic TMDs and whose FAI score was 20 and above. The control group consisted of individuals whose FAI score was 15 and below. The study group consisted of 42 individuals diagnosed with myogenic TMDs, and the control group consisted of 42 individuals who had not experienced any pain related to the TMJ in the last year.

### 2.4 Assessment of Pain Severity

The pain intensity assessment was conducted using the Visual Analog Scale (VAS), which is shown to be valid and reliable [22, 23]. Participants were asked to indicate the severity of pain they had experienced in the jaw and neck joints at rest in the last 1 week on a 100 mm long line positioned horizontally. The marked points were measured with a ruler and recorded in millimetres (mm).

### 2.5 Symptom Severity and Assessment of Disability

Fonseca Anamnestic Index (FAI) used to assess the severity of TMDs, which is reported to be valid and reliable in the Turkish population [20]. FAI, which evaluates the severity of TMDs according to signs and symptoms, is a simple‐to‐use questionnaire consisting of 10 specific questions with 3 answer options (yes–sometimes–no) [24]. In the questionnaire scoring, the “yes” option is scored as 10 points, the “sometimes” option as 5 points and the “no” option as 0 points. 0–15 points obtained as a result of the questionnaire: No TMDs, 20–40 points: Mild TMDs, 45–65 points: Moderate TMDs, 70–100 points: Refers to the presence of severe TMDs [24].

The 8‐item short form of the Jaw Function Limitation Scale (JFLS‐8), which is reported to be valid and reliable, was used to assess jaw function and limitation [25]. Eight items in the survey are rated as 0 Points: “no restrictions” and 10 Points: “Severe Restrictions” [26]. The total score varies between 0 and 80, and it is reported that higher scores indicate a serious level of limitation [25].

Neck Disability Index (NDI), which has Turkish validity and reliability, was used to determine the level of neck‐related disability [27]. The 10‐item questionnaire, the first four items of which are related to subjective symptoms and the other six items are related to activities of daily living, is evaluated as 0 points no disability, 5–14 points mild disability, 15–24 points moderate disability, 25–34 points severe disability, and above 35 points complete disability [28, 29].

### 2.6 Assessment of Temporomandibular Joint ROM

Individuals' ROM (ROM) of the TMJ was evaluated using the TheraBite ROM Scale (TheraBite, Platon Medical Ltd., United Kingdom). Similar to previous studies [30, 31], the measurements were performed with the subjects in a comfortable supine position and with the neck in a neutral position. The individuals were asked to open their mouths first to the pain limit and then to the fullest extent they could. The distance between the mandibular and maxillary incisors was measured with the TheraBite ROM Scale. Joint ROM measurements were repeated three times, and the average values of these measurements were recorded in millimetres [31].

### 2.7 Assessment of Cervical Joint Position Sense

**FIGURE 1:**
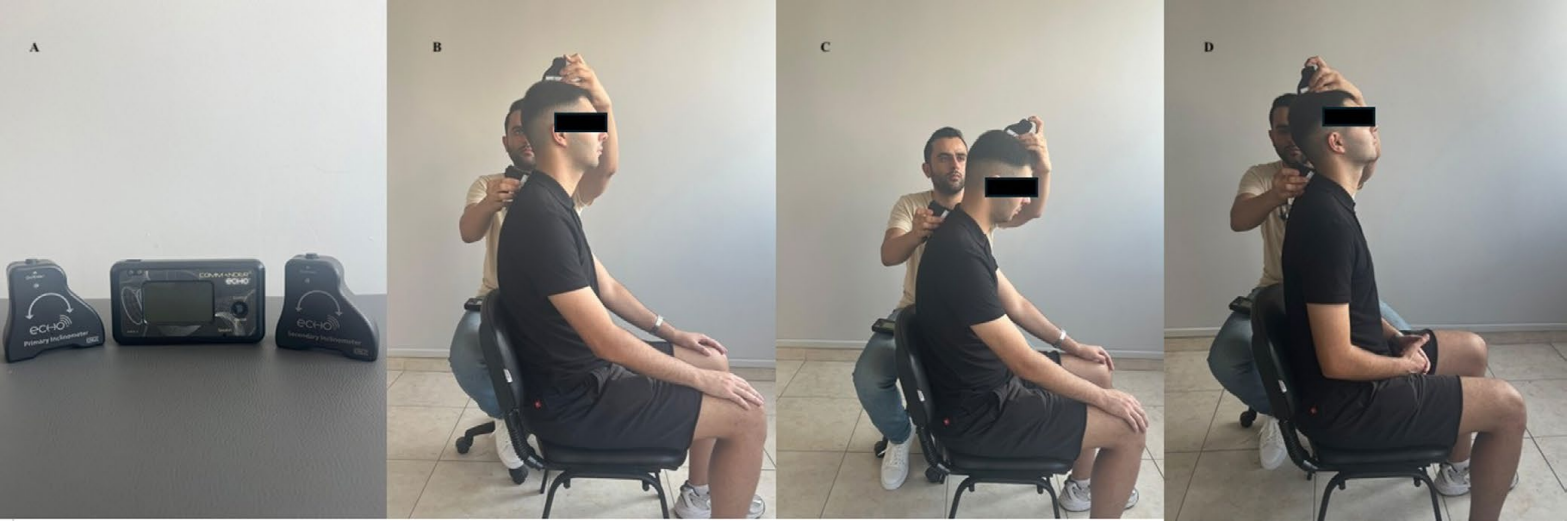
(A) Digital inclinometer, (B) cervical joint position sense starting position (neutral), (C) target angle test final position (flexion), (D) target angle test final position (extension).

The participants' cervical joint position sense was evaluated using the target angle test with the help of a digital inclinometer (JTECH Medical, Midvale, UT, USA), which is reported to be valid and reliable [32, 33]. Assessments were made with the individuals sitting on a chair, hips and knees flexed at 90° and eyes closed [32, 33]. Target angle tests to evaluate joint position sense were performed in 30° neck flexion and 20° neck extension positions. The heads of the individuals who were in the sitting position with their eyes closed were brought from the neutral position to the target angle by the assessor and the individuals were asked to keep this position in their minds. After learning the target position, the individuals whose heads were placed in a neutral position were asked to move their heads to the target angle. The angular difference between the target angle and the final position was recorded in degrees. The tests were repeated three times with an interval of 30 s and the average of these measurements was recorded (Figure 1).

### 2.8 Assessment of Head Posture

The head posture of the individuals was evaluated with PostureScreen Mobile (PSM) (PostureCo, Inc., Trinity, Florida, USA) application, which has recently been reported to be valid and reliable on devices with iOS and Android software [34, 35]. The application, which offers the opportunity to evaluate the static posture of individuals in standing posture anteriorly, posteriorly and laterally, reports numerical deviations from normal posture [34]. Following the capture of photographs in the anterior, posterior, and lateral directions using a device equipped with Android software by the individuals included in the study, the reference points determined by the application were marked on the touchscreen. Deviation values measured relative to these reference points via the software were recorded in centimetres or degrees.

### 2.9 Statistical Analysis

The data of the study were analysed with the statistical program (Statistical Package for Social Science for Windows, Version 26.0, Inc., an IBM Company, Chicago, IL, USA). The conformity of the variables evaluated in the study to normal distribution was assessed using visual (histograms, probability graphs) and analytical methods (Kolmogorov–Smirnov test). Since the variables are not normally distributed, the findings are presented as median (interquartile range). For categorical variables, the difference between groups was analysed by chi‐square test. Differences in parameters between groups were examined with the Mann–Whitney *U* test. The relationship between the deviation in CJPS and changes in head position and the ROM of the jaw joint, pain intensity and the level of limitation in jaw function were analysed using Spearman's test. For all statistical analysis methods, the significance level was accepted as a *p* value less than 0.05.

## 3 Results

There was no significant difference between the groups in terms of age (*p* = 0.231), height (*p* = 0.897) and body weight (*p* = 0.239) (Table 1).

**TABLE 1:** Demographic characteristics of groups.

Parameters	Control groups (*n* = 42)	Study group (*n* = 42)	*p*
Age (years)	20 (19–22)	21 (20–23)	0.231
Height (m)	1.68 (1.63–1.74)	1.69 (1.60–1.75)	0.897
Body mass (kg)	62.5 (55.5–70.2)	63.0 (57.7–75.5)	0.239
Body mass index (kg/m^2^)	21.5 (19.9–23.3)	22.9 (20.7–25.8)	0.047
Sex (*n*/%)			
Male	11 (26.2%)	10 (23.8%)	0.801
Female	31 (73.8%)	32 (76.2%)	

The severity of pain experienced by individuals diagnosed with TMDs in the neck and jaw joints was found to be higher compared to the control group (*p* < 0.001). FAI, JFLS‐8 and NDI scores of individuals diagnosed with TMDs were higher than the control group (*p* < 0.001). TMJ ROM values measured up to the pain limit and maximally were found to be lower in individuals with TMDs compared to the control group (*p* < 0.001) (Table 2).

**TABLE 2:** Median (interquartile range) of parameters assessed in both groups.

Parameters	Control groups (*n* = 42)	Study group (*n* = 42)	*p*
Visual Analog Scale—TMJ (mm)	0 (0–60)	49.5 (28.5–63)	**< 0**.**001** *
Visual Analog Scale—cervical (mm)	0 (0–10)	56 (28.2–70)	**< 0**.**001** *
Fonseca Anamnestic Index (score)	10 (5–15)	60 (45–70)	**< 0**.**001** *
Jaw Functional Limitation Scale‐8 (score)	0 (0–3.25)	17.50 (7.75–25.5)	**< 0**.**001** *
Neck Disability Index (score)	2 (1–5)	12 (8.75–17)	**< 0**.**001** *
TMJ ROM (up to the pain limit‐mm)	47 (40–53.25)	34 (26–41)	**< 0**.**001** *
TMJ ROM (maximum ROM‐mm)	49 (42.5–53.25)	42 (37.75–46)	**< 0**.**001** *

Abbreviations: ROM, range of motion; TMJ, temporomandibular joint.

* *p* < 0.05, Mann–Whitney *U* test.

The angular deviation values in the flexion and extension directions of the target angle test performed to evaluate the CJPS were found to be higher in individuals with TMDs compared to individuals in control group (*p* < 0.001). There was no statistically significant difference between the groups in terms of head posture analyses performed anteriorly, laterally and posteriorly (*p* > 0.05) (Table 3).

**TABLE 3:** Median (interquartile range) of neck position sense and head posture evaluated in both groups.

Parameters	Control group (*n* = 42)	Study group (*n* = 42)	*p*
Error for target angle test (flexion°)	2 (1.30–3.15)	4.15 (2.52–6.07)	**< 0**.**001** *
Error for target angle test (extension°)	1.80 (1–3)	3.30 (2–4.60)	**< 0**.**001** *
Head anterior translations (cm)	0.51 (0.23–0.77)	0.47 (0.21–0.88)	0.961
Head anterior angulations (°)	0 (0–2.55)	0 (0–2.71)	0.262
Head right lateral translations (cm)	3.53 (2.04–4.98)	3.51 (2.83–4.78)	0.865
Head right lateral angulations (°)	11.26 (6–15.46)	11.70 (8.36–15.23)	0.883
Head posterior translations (cm)	0.75 (0.53–1.09)	0.83 (0.22–1.20)	0.619
Head posterior angulations (°)	0 (0–0.52)	0 (0–2.65)	0.156
Head left lateral translations (cm)	3.73 (2.58–5.25)	3.85 (2.73–5.13)	0.758
Head left lateral angulations (°)	10.81 (7.33–15.60)	12.52 (8.60–15.99)	0.474

* *p* < 0.05, Mann–Whitney *U* test.

In individuals with TMDs, no relationship was observed between changes in head position and jaw joint movement range, pain and dysfunction severity, and level of limitation in jaw functions (*p* > 0.05) (Table 4).

**TABLE 4:** Relationship between head posture and other parameters in individuals with TMDs.

Variables	VAS‐TMJ	VAS‐cervical	FAI score	NDI score	JFLS‐8 score	TMJ ROM At pain limit	TMJ ROM
Anterior head translation	−0.103	−0.194	−0.033	−0.202	−0.078	0.260	0.061
Anterior head angulation	0.053	−0.257	−0.185	−0.086	0.195	−0.127	−0.157
Right lateral head translation	0.097	0.118	0.201	0.169	0.077	0.087	−0.049
Right lateral head angulation	0.090	0.158	0.163	0.090	0.046	0.124	−0.043
Posterior head translation	−0.061	−0.165	−0.024	−0.121	−0.120	−0.257	−0.247
Posterior head angulation	0.207	−0.058	−0.008	0.161	0.241	0.136	0.053
Left lateral head translation	0.358*	0.291	0.267	0.272	0.118	−0.279	−0.125
Left lateral head angulation	0.294	**0**.**308** *	0.269	0.256	0.116	**−0**.**324** *	−0.132

Abbreviations: FAI, Fonseca Anamnestic Index; JFLS, Jaw Functional Limitation Scale; NDI, Neck Disability Index; ROM, range of motion; TMJ, temporomandibular joint; VAS, Visual Analog Scale.

* Spearman correlation is significant at the 0.05 level (two‐tailed).

Correlation analyses show that VAS‐TMJ was correlated with VAS‐cervical (*r* = 0.716, *p* < 0.001), FAI (*r* = 0.759, *p* < 0.001), NDI (*r* = 0.643, *p* < 0.001) and JFLS‐8 (*r* = 0.778, *p* < 0.001) scores. VAS‐TMJ had significant negative correlation at fair to moderate strength with maximum TMJ ROM (*r* = −0.332, *p* < 0.001) and TMJ ROM at pain limit (*r* = −0.458, *p* < 0.001). VAS‐cervical scores had moderate to good correlation with JFLS‐8 (*r* = 0.584, *p* < 0.001), FAI (*r* = 0.664, *p* < 0.001), NDI (*r* = 0.694, *p* < 0.001) and negative fair correlation with TMJ ROM at pain limit (*r* = −0.279, *p* < 0.05). FAI scores shown to have good positive correlation with NDI (*r* = 0.648, *p* < 0.001) and JFLS‐8 (*r* = 0.709, *p* < 0.001) scores. FAI scores had fair to moderate negative correlation with TMJ ROM at pain limit (*r* = −0.502, *p* < 0.001) and maximum TMJ ROM (*r* = −0.389, *p* < 0.001) values. Results showed good positive correlation between NDI and JFLS‐8 scores (*r* = 0.677, *p* < 0.001) also fair negative correlation between NDI and TMJ ROM values at pain limit (*r* = −0.258, *p* < 0.05). JFLS‐8 exhibited negative fair to moderate relationship with TMJ ROM values at pain limit (*r* = −0.479, *p* < 0.001) and maximum TMJ ROM (*r* = −0.353, *p* < 0.001) (Table 5). No significant relationship was found between neck position sense and head posture values (*p* > 0.05).

**TABLE 5:** Relationship between evaluated parameters in individuals in TMDs.

Variables	Neck position sense extension	VAS–TMJ	VAS‐cervical	FAI score	NDI score	JFLS‐8 score	TMJ ROM at pain limit	TMJ ROM
Neck position Sense‐Flexion	**−0**.**458** **	−0.092	0.240	0.023	0.264	−0.160	0.170	0.260
Neck position sense extension		0.114	−0.036	−0.134	0.136	0.209	0.089	0.015
VAS‐TMJ			**0**.**716** **	**0**.**759** **	**0**.**643** **	**0**.**778** **	**−0**.**458** **	**−0**.**332** **
VAS‐cervical				**0**.**664** **	**0**.**694** **	**0**.**584** **	**−0**.**279** *	−0.162
FAI score					**0**.**648** **	**0**.**709** **	**−0**.**502** **	−**0**.**389** **
NDI score						**0**.**677** **	**−0**.**258** *	0.193
JFLS‐8 score							**−0**.**479** **	**−0**.**353** **
TMJ‐ROM at pain limit								**0**.**782** **

Abbreviations: VAS, Visual Analog Scale; TMJ, temporomandibular joint; FAI, Fonseca Anamnestic Index; NDI, Neck Disability Index; JFLS, Jaw Functional Limitation Scale; ROM, range of motion.

* Spearman correlation is significant at the 0.05 level (two‐tailed).

** Spearman correlation is significant at the 0.01 level (two‐tailed).

## 4 Discussion

This research is the first to evaluate the head posture and CJPS of individuals with TMDs and to compare them with controls. The use of objective measurement methods in the evaluation of jaw joint ROM, CJPS and head posture is another strength of the study. It was found that in individuals diagnosed with TMDs, in addition to the severity of pain in the neck and jaw joints, FAI, JFLS‐8 and NDI scores were higher than those in the control group. The results of our study show that subjectively reported pain intensity localised to the cervical and temporomandibular joints is higher in individuals with TMDs compared to the control group. Previous research, showing parallels with our study results, demonstrates a close functional relationship between the neck joint and TMJ, and a higher prevalence of neck pain in individuals with TMJ compared to healthy controls [36, 37]. Moreover, the results of a study investigating the effect of TMDs on mandibular mobility in young adults, which was similar to our study in terms of participant age, reported that the mean severity of jaw joint pain in individuals with TMDs was 6 out of 10 and the pain severity was higher compared to the control group [38].

The normal amount of mouth opening in adults is expected to average 45–50 mm when measured between the upper and lower incisors [39]. The maximal and pain limit TMJ ROM values of the individuals in the control group of our study are compatible with the norm values. The results of a systematic review‐meta‐analysis study evaluating the range of jaw motion in adults with persistent TMDs show that 24 out of 38 studies measuring active maximum mouth opening were lower in individuals with TMDs compared with the control group [39]. In the same study, it was stated that the amount of ROM decreased by 4.65 mm in individuals with TMDs compared to controls, while this value was recorded as 3.28 mm in adults diagnosed with myogenic TMDs, a subgroup of TMDs [40]. Wolan‐Nieroda et al. [38], on the other hand, report that the mobility of the mandible in all planes is significantly lower in patients with TMDs compared to healthy individuals. The ROM values of the individuals in the study group diagnosed with myogenic TMDs were lower than the control group. Our correlation analyses show that decrease in TMJ ROM were related to increase in disability and pain of cervical region and TMJ. Studies examining the relationship between TMJ and the cervical region are in parallel with the results of our study, which revealed TMJ ROM is associated with high NDI scores and increased cervical joint pain severity in individuals with TMDs [6, 9, 38].

The results of our study indicate that individuals diagnosed with myogenic TMDs have higher deviations in cervical joint position sensation compared to individuals in the control group. Furthermore, it was found that neck disability was higher in patients with TMDs than controls; however, deviations in cervical joint position sensation did not correlate with severity of TMJ pain and disability. Reduced sense of joint position may be related to increased neck disability in patients with TMDs. Previous studies have shown a relationship between pain and impaired joint position sense, and it may be hypothesized that neck and jaw‐related pain experienced by patients with TMD may cause impaired cervical joint position sense [41, 42]. On the other hand, reduced cervical joint position sensation has a potential to chance the jaw function because of neural, anatomical and biomechanical interaction between neck and jaw. Considering that chewing activity is provided by orofacial sensorimotor and cervical system integration and that appropriate sensory inputs from these systems provide jaw and orofacial muscle coordination, impaired cervical joint position sense may cause abnormal cervical posture during functional activities [7, 8, 43]. During functional activities such as chewing and speaking, the neck muscles, especially the sternocleidomastoid muscle, coactivate to stabilise the head and also respond to the increased workload along with the masseter muscle. Abnormal cervical posture during these activities may alter jaw movement and masticatory muscle activation [43, 44]. These changes may be a cause of jaw‐related pain or TMDs over time. Therefore, evaluation of cervical joint position sense in patients with TMDs and use of approaches improving proprioception such as balance and exercise in patients with impairment of cervical joint position sense may be useful in the control of TMD‐related symptoms.

FHP, which is among the risk factors for TMDs, has the potential to affect the position of the centre of gravity and confirms the relationship between body posture and TMDs [6]. Similarly, postural changes in the cervical region can cause TMDs, altering the orientation of the head and thus the mandibular position [6]. Previous studies show that there is a relationship between TMDs and posture and that individuals with TMDs usually exhibit an excessive FHP with shortening of the posterior cervical extensor muscle group [6, 10, 11]. The correlation results in this study support this relationship between the cervical region and the jaw joint in that an increase in the severity of pain and dysfunction in the cervical region increases the severity of pain in the jaw joint and decreases the ROM. Moreover, the relationship between VAS‐Cervical and FAI and JFLS‐8 scores shown in our study suggests that avoidance of parafunctional behaviours specific to the jaw joint, patient education and activity modifications may be effective in cervical pain management.

Consistent with the results of our study, there are many studies indicating that postural angular deviations are not associated with neck disability, jaw limitation and pain severity [45, 46, 47]. Armijo‐Olivo et al. [45] reported a statistically significant difference in cranio‐cervical posture between patients with myogenic TMDs and healthy individuals but stated that this difference was not clinically significant. No statistically significant difference was found by the present study in terms of cranio‐cervical posture. Furthermore, head posture parameters did not correlate with severity of TMJ pain and disability. The fact that results do not establish a significant relationship between head and neck posture and TMDs, is consistent with other studies evaluating head and neck posture using photographic analysis methods [46, 47]. Studies included in a systematic review examining the relationship between TMDs and head and neck posture show that 30%–42% of them demonstrate no relationship between TMDs and head and neck posture [48]. On the other hand, in the same study, it was stated that the relationship between TMDs and head and neck posture is still controversial and uncertain [48]. Our study did not account for the factors that could potentially affect cervical joint position and head posture, such as lifestyle or postural habits. In future studies, considering these factors would be beneficial for better understanding their impact on cervical joint position sensation, head posture and TMDs, as well as the potential interactions between these factors.

The study has a few limitations. First, this study was planned as a cross‐sectional study therefore longitudinal studies are needed to establish a causal relationship between TMDs and head and cervical posture. Second, clinical measurements such as intervertebral physiological and accessory movements to explore clinical patterns was not assessed in the present study. If these evaluations could be made, the relationships between neck dysfunction and temporomandibular disorders could be better demonstrated. Finally, our study covers a limited age group. Therefore, it is necessary to conduct research targeting older age groups to consider degenerative changes in the cervical area.

## 5 Conclusion

Compared to controls, individuals with TMDs demonstrate significantly greater angular deviations in CJPS during both flexion and extension movements. Additionally, patients with TMDs exhibit increased TMJ pain, restricted ROM and functional impairments compared to the control group. However, no significant differences in head posture alterations were observed between the two groups. In addition, the high correlations between the parameters related to the neck and TMJ reinforce the evidence regarding interaction between these two regions. Our study suggests that clinical strategies targeting balance and proprioception, alongside functional neck interventions, may enhance TMJ functionality in individuals with TMDs.

## Author Contributions

Conceptualization: Ümit Yüzbaşioğlu, Serkan Taş. Methodology: Ümit Yüzbaşioğlu, Besime Ahu Kaynak, Serkan Taş. Formal analysis and investigation: Ümit Yüzbaşioğlu, Besime Ahu Kaynak, Serkan Taş. Writing – original draft preparation: Ümit Yüzbaşioğlu, Serkan Taş. Writing – review and editing: Ümit Yüzbaşioğlu, Serkan Taş.

## Conflicts of Interest

The authors declare no conflicts of interest.

### Peer Review

The peer review history for this article is available at https://www.webofscience.com/api/gateway/wos/peer‐review/10.1111/joor.13885.

## Supporting information


Data S1.

## Data Availability

The data are not publicly available due to privacy or ethical restrictions.
